# Jin-Zhen oral liquid for pediatric coronavirus disease (COVID-19): A randomly controlled, open-label, and non-inferiority trial at multiple clinical centers

**DOI:** 10.3389/fphar.2023.1094089

**Published:** 2023-02-27

**Authors:** Qian Dong, Hongmei Qiao, Huiyi Jiang, Lixiao Liu, Yanling Ge, Fang-Jiao Zong, Yanan Li, Bingzi Dong, Sujuan Hu, Dongmei Meng, Rong Jin, Xiangshi Wang, Hailing Chang, Xiaolong Xu, Chenjing Wang, Yu Cao, Han-Ting Zhang, Qingquan Liu

**Affiliations:** ^1^ Children’s Medical Center, The Affiliated Hospital of Qingdao University, Qingdao, China; ^2^ Department of pediatric respiratory medicine, The First Hospital of Jilin University, Changchun, China; ^3^ Eastern Division of Pediatrics, The First Hospital of Jilin University, Changchun, China; ^4^ Department of pediatrics, Shanghai Pudong Hospital, Shanghai, China; ^5^ Department of Infection Diseases, Children’s Hospital of Fudan University, Shanghai, China; ^6^ Department of Pharmacology, Qingdao University School of Pharmacy, Qingdao, China; ^7^ Emergency Department, Beijing hospital of Traditional Chinese Medicine, Capital Medical University, Beijing, China

**Keywords:** traditional Chinese medicine, Jin-Zhen oral liquid, pediatric COVID-19, randomized controlled clinical trial, multiple clinical centers

## Abstract

**Background:** As the coronavirus disease 2019 (COVID-19) pandemic progressed, especially with the emergence of the Omicron variant, the proportion of infected children and adolescents increased significantly. Some treatment such as Chinese herbal medicine has been administered for COVID-19 as a therapeutic option. Jin-Zhen Oral Liquid is widely used for pediatric acute bronchitis, while the efficacy and safety in the treatment of pediatric COVID-19 are unclear.

**Methods:** We conducted a randomized controlled, open-label, multicenter, non-inferiority clinical study involving hospitalized children with mild to moderate COVID-19. Children eligible for enrollment were randomly assigned in a 1:1 ratio to Jin-Zhen Oral Liquid (the treatment group) and Jinhua Qinggan Granules (the positive control group) and received the respective agent for 14 days, followed by a 14-day follow-up after discontinuation of the treatment. The primary efficacy endpoint was the time to first negative viral testing. The secondary endpoints were the time and rate of major symptoms disappearance, duration of hospitalization, and the proportion of symptoms changed from asymptomatic or mild to moderate or severe/critical illness. In addition, the safety end points of any adverse events were observed.

**Results:** A total of 240 child patients were assigned randomly into the Jin-Zhen Oral Liquid (117 patients) and Jinhua Qinggan Granules (123 patients) groups. There was no significant difference of the baselines in terms of the clinical characteristics and initial symptoms between the two groups. After 14-day administration, the time to first negative viral testing in the Jin-Zhen group (median 6.0 days, 95% CI 5.0-6.0) was significantly shorter compared with the positive control Jinhua Qinggan Granules group (median 7.0 days, 95% CI 7.0-8.0). The time and rate of major clinical symptoms disappearance were comparable to the positive control. The symptom disappearance time of pharyngalgia and hospitalization duration were significantly shortened in the Jin-zhen Oral Liquid group. No participants in either group experienced post-treatment exacerbation to severe or critical illness. No adverse events were observed in the Jin-Zhen Oral Liquid treatment group (0.0%) while 1 patient with adverse events occurred in the positive control Jinhua Qinggan granules group (0.8%). No serious adverse events were observed during the study period in both groups.

**Conclusion:** Jin-Zhen Oral Liquid is safe and effective in the treatment of mild to medium COVID-19 in children. It is non-inferior to Jinhua Qinggan granules in shortening the time to first negative viral testing, the time and rate of major clinical symptoms disappearance, and the hospitalization duration. The results suggest that Jin-Zhen Oral Liquid can be a recommended drug for treatment of pediatric COVID-19 patients.

## Introduction

The coronavirus infected pneumonia (COVID-19) is caused by severe acute respiratory syndrome coronavirus 2 (SARS-CoV-2) and is a serious threat to public health. It can invade multiple organs, and the severe cases can rapidly develop acute respiratory distress syndrome and multi-organ failures. As the COVID-19 pandemic progresses, especially with the emergence of the Omicron variant, the proportion of children infected significantly increased. By early 2022, pediatric patients accounted for 25% of cases, higher than the <2% of infection rate in the early years of previous pandemic (Children and COVID-19, 2022). Although highly infectious, COVID-19 pneumonia is less symptomatic in children than in adults, with a large proportion of asymptomatic infections but lower rate of hospitalization and mortality. However, for pediatric COVID-19 patients with comorbidities, severe and critical cases may still occur (Alsohime et al., 2020). Therefore, early treatment is important for preventing of disease progression and accelerating recovery of COVID-19 in children.

Currently, the US FDA approves the oral antiviral drug nimatoprevir/ritonavir (Paxlovid^®^) for adults and children with mild and moderate COVID-19 that may progress to severe cases (FDA), and also approves ramucir (Veklury^®^) for adult and child patients with COVID-19 requiring hospitalization (NIH, 2022). Both are expensive and the latter requires intravenous administration. Thus, those factors limit the administration to children with mild to moderate infections. Development of an agent which is easy to administer, cost-effective, and highly effective is a major challenge. Chinese herbal medicine is widely administrated as a therapeutic tool for COVID-19 during these years. Traditional Chinese medicine (TCM) has unique advantages in mobilizing the body’s resistibility and homeostasis, improving the clinical symptoms, reducing complications, and improving the quality of life (National Health Commission and Medicine NAoTC, 2022).

Jin-Zhen Oral Liquid is developed based on the traditional clinical experience formula “Lingyang Qingfei San”, which consists of *Cornu caprae hircus* (Shan yang jiao)*, Thunberg Fritillary Bulb* (Ping bei mu)*, Chinese rhubarb* (Da huang,)*, Scutellariae Radix* (Huang qin)*, Chloriti Lapis* (Qing meng shi)*, Red Paeony Root* (Shi gao)*, Artificial Bezoar* (Rengong niu huang)*,* and *Glycyrrhizae Radix* (Zhi gan cao) (Liu et al., 2014). It is used for pediatric acute bronchitis with phlegm-heat cough (the main clinical manifestations such as fever, cough, yellow sputum, red tongue, yellow greasy coating of tongue) (Tao et al., 2020). Jin-Zhen Oral Liquid show remarkable clinical efficacy, safety and reliability, with ease of administration and high compliance for children. It has been recommended by several guidelines and consensus recommendations including hand foot and mouth disease (HFMD) and *mycoplasma* pneumonia in children. In addition, *in vitro* and *in vivo* studies have shown that Jin-Zhen granule could inhibit the cytopathic effect of COVID-19 infection on Vero E6 cells. Jin-Zhen granule significantly reduce the viral load in the lung tissue of infected mice. It also exhibits anti-SARS-COV-2 activity when administered 2 h after the cells were infected (Ma et al., 2022). In addition, Jin-Zhen granule dose-dependently ameliorates the levels of cellular inflammatory factors IL-1α, IL-6, MIP-1β, and CCL-5 to regulate the cellular inflammatory response caused by SARS-COV-2 (Ma et al., 2022). Those studies suggest that Jin-Zhen Oral Liquid has anti-COVID-19 effect on both prophylactic and therapeutic administration.

To provide new clinical evidence for the treatment of COVID-19 in children, we evaluated the efficacy of Jin-Zhen Oral Liquid in shortening the time to first negative viral testing, reducing hospitalization duration and improving symptoms in pediatric COVID-19 patients. We observed the non-inferiority efficacy and safety of Jin-Zhen Oral Liquid in the treatment of pediatric COVID-19 and compared to the established positive control treatment Jinhua Qinggan granules.

## Methods

### Study design

In this randomized controlled, multicenter, open-label, positive controlled, non-inferiority clinical trial, we evaluated the efficacy of Jin-Zhen Oral Liquid in the treatment of Chinese pediatric COVID-19 patients. To evaluate the efficiency and safety of Jin-Zhen Oral Liquid, Jinhua Qinggan granules were selected as the positive control therapy to be compared with the non-inferiority. Jinhua Qinggan granules have been reported to relieve the clinical symptoms and improve prognosis in the confirmed and suspected COVID-19 adult patients and was recommended as a therapy agent in the Guidelines on Diagnosis and Treatment Protocol of novel coronavirus pneumonia (COVID-19) in China (An et al., 2021; National Health Commission and Medicine NAoTC, 2022). This trial was conducted at four clinical centers in China (The Affiliated Hospital of Qingdao University, The First Hospital of Jilin University, Shanghai Pudong Hospital, and Children’s Hospital of Fudan University). Ethics committee approval was obtained from all centers. The trial was conducted in compliance with the Declaration of Helsinki and the International Conference of Harmonization-Good Clinical Practice (ICH-GCP). The study is available at www.clinicaltrials.gov registration number: NCT05507489.

### Patients

The diagnostic criteria of COVID-19 were in accordance with the Guideline on Diagnosis and Treatment Protocol for COVID-19 (Version 9) (National Health Commission and Medicine NAoTC, 2022). All the patients recruited in this trial were in hospital from four clinical centers. Patients enrolled in this study met the following inclusion criteria: 1) patients between the ages 3–18 years old; 2) positive COVID-19 nucleic acid test or positive for both SARS-CoV-2-specific IgM antibodies and IgG antibodies in those patients who were not vaccinated against COVID-19; 3) asymptomatic infected or clinically typed as mild-moderate symptoms (with or without visible imaging change detected by computed tomography (CT) scan).

Exclusion criteria included (1) meeting early warning indicators of severe/critical type (including increased respiratory rate, poor mental response, lethargy, progressive elevation of lactate, significant elevation of inflammatory and infective factors including C-reactive protein (CRP), procalcitonin (PCT), ferritin, imaging showing bilateral or multiple lung lobes with inflammatory infiltration, pleural effusion, or rapid progression of lesions within a short period of time), patients with underlying diseases including congenital heart disease, bronchopulmonary dysplasia, airway malformations, severe malnutrition, allergic asthma (undertake Salmeteroticasone inhalation administration), immunodeficiency or usage of immunosuppressants, chronic kidney disease, liver dysfunction, endocrine diseases, autoimmune diseases, malignant tumors, gastrointestinal tract disorders that may affect the patients’ participation in the trial or the outcome of the study, and patients with allergies (National Health Commission and Medicine NAoTC, 2022); (2) children who used the same type of proprietary Chinese medicine for more than 3 days before enrollment.

### Study medication

Jin-Zhen Oral Liquid (State Food and Drug Administration, SFDA approval No. Z10970018, Kanion Pharmaceutical (Jiangsu) Co., Ltd. Specification 10ml/bottle) is brownish red to brownish yellow liquid, slightly fragrant, and slightly bitter in taste. Jin-Zhen Oral Liquid mainly contains *Cornu caprae hircus* (Shan yang jiao)*, Thunberg Fritillary Bulb* (Ping bei mu)*, Chinese rhubarb* (Da huang)*, Scutellariae Radix* (Huang qin), *Chloriti Lapis* (Qing meng shi)*, Red Paeony Root* (Shi gao), *Artificial Bezoar* (Rengong niu huang), and *Glycyrrhizae Radix* (Zhi gan cao). Jinhua Qinggan granules (SFDA approval No. Z20160001, Juxiechang (Beijing) Pharmaceuticals Co., Ltd., Specification 5g/bag), the positive control administration, is light brown to brown in color, slightly fragrant, and bitter in taste. Jinhua Qinggan granules contains *Honeysuckle* (Jin yin hua), *Red Paeony Root* (Shi gao), *Ephedrae Herba* (Ma huang)*, Glycyrrhizae Radix* (Zhi gan cao)*, Armeniacae Semen* (Xing ren), *Artemisia annua* (Qing hao)*, Scutellariae Radix* (Huang qin)*, Fructus Forsythiae* (Lian qiao)*, Thunberg Fritillary Bulb* (Zhe bei mu)*, Rhizoma Anemarrhenae* (Zhi mu)*, Arctii Fructus* (Niu bang zi)*,* and *Menthae Haplocalycis Herba* (Bo he). Both medication quality conforms the national medication standard established by the SFDA. All the participants administrated with Western medicine treatment according to the recommendation of the Diagnosis and Treatment Protocol for COVID-19 (Version 9) (National Health Commission and Medicine NAoTC, 2022).

### Study procedure

**FIGURE 1:**
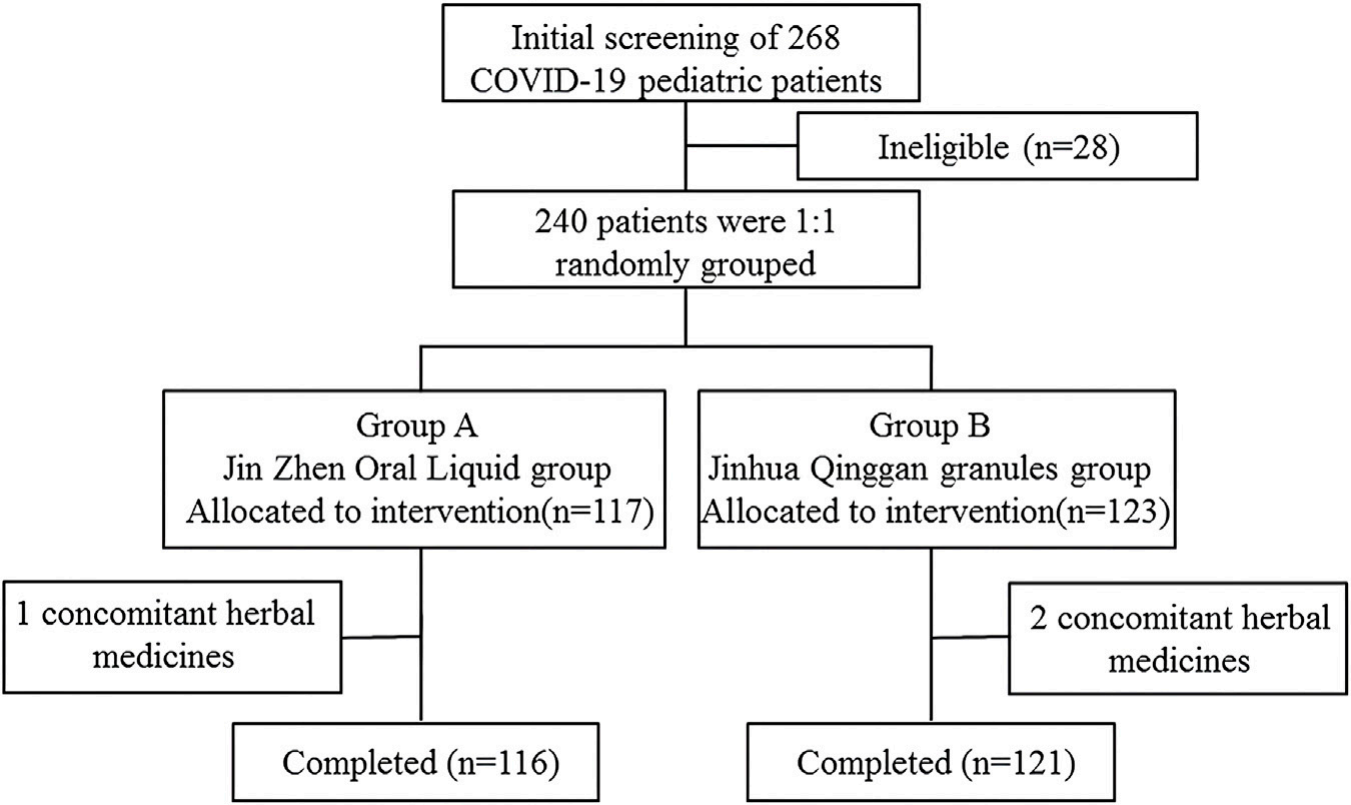
Flowchart of screening, randomization, and treatment of subjects.

A total of 240 participants were enrolled, and eligible children were randomly assigned (1:1) to the Jin-Zhen Oral Liquid group (treatment group) and the Jin Hua Qinggan Granules group (positive control group) (Figure 1). Jin-Zhen Oral Liquid was administered orally, 10 ml twice a day for children aged 3 years or younger, and 10 ml three times a day for children aged 4–7 years old, and 15 ml three times a day for children aged 8–18 years. The administration of Jinhua Qinggan Granules was 5 g three times per day for children aged 8–18 years old, or 2.5 g three times per day for children aged 3–7 years. Children were treated for 14 days (the drug could end earlier if they reached the discharge criteria) and were followed up on the 1st, 2nd, 3rd, 4th, 5th, 6th, 7th, 10th, 14th and 28th days of enrollment. According to the Guideline on Diagnosis and Treatment Protocol for COVID-19 (Trial Version 7), criteria of discharge included 1) Body temperature recovers to normal range for more than 3 days; 2) Symptoms of respiratory system improved significantly; 3) Imaging examination such as CT scan of lung showed significant improvement in acute exudative lesions; 4) Both of the Ct values of the N gene and the ORF gene of the nucleic acid detection of SARS-CoV-2 were <35 (the cutoff value is 40, and the sampling time was at least 24 h apart), or two consecutive negative viral testing of the SARS-CoV-2 nucleic acid (the PCR cutoff value ≥ 35 and the sampling time is at least 24 h apart). The viral testing of nucleic acid was performed using nasal or pharyngeal swabs daily and detected by the clinical laboratory in each clinical center. The last follow-up visit was conducted on the 14th day after discontinuation of the drug if the treatment was ended earlier. Baseline data and concomitant medications were collected during the screening period, and combined medications, disease progression, vital signs, blood samples, and nasopharyngeal swabs were collected during the follow-up period. The final follow-up visit was conducted by telephone or outpatient clinic, with the inquiry about the basic condition of the child and whether any adverse events had occurred.

During the observation period, the use of other herbal medicines for the treatment of COVID-19 in children, including herbal soup, granules or Chinese medicines is limited.

### Primary and secondary end points

The primary endpoint was the time to first negative viral testing of COVID-19. Secondary endpoints were the time and rate of major clinical symptoms disappearance, including fever, dry cough, sputum, fatigue, vomiting, diarrhea, pharyngoxerosis, pharyngalgia, nasal congestion, and nasal discharge, the duration of hospitalization (i.e., the time from medication admission to the time of discharge when met the discharge criteria), and the proportion of symptoms changed from asymptomatic or mild to moderate or to severe/critical illness.

### Safety monitoring

Before and after treatment, all participants were assessed for symptoms, temperature, safety, medication compliance, and adverse events (AEs). Safety outcomes include the incidence of adverse events, vital signs (blood pressure, heart rate, respiration, temperature, *etc.*), and laboratory tests (blood routine test, urine routine test, liver and renal function). Investigator inquired the participants about the occurrence of AEs at each follow-up visit, and recorded the time of onset and end, duration, severity, administration, and regression of AEs. Severity was graded in accordance with the Common Terminology Criteria for Adverse Events (NCI-CTCAE V5.0) developed by the National Cancer Institute of the National Institutes of Health of the US Department of Health and Human Services. The investigators assessed the possible association between AEs and the study drug. 

### Randomization

All participants were randomly divided into the Jin-Zhen Oral Liquid group and the Jinhua Qinggan granules group (as the positive control group) at the ratio of 1:1. Randomization of subjects was conducted by block randomization, and the random hiding used the random envelopes. The random numbers were generated by block randomization method and assigned using SAS statistical analysis software (Version 9.4). This study was designed as an open-label trial. Data analysis was performed independently by professional statisticians.

### Statistical analysis

The statistical analysis was performed using SAS 9.4 software. The data are presented as means ± standard deviation (SD) and were tested using a two-way test with α = 0.05. Comparisons between groups were performed using *t*-test, or Wilcoxon rank test as appropriate.

In this study, the non-inferiority was analyzed in the full analysis set (FAS), including all the participants underwent randomization, took at least once administration of Jin-Zhen Oral Liquid or Jinhua Qinggan granules, and recorded the visit at least once. The sensitivity analysis was performed in per protocol set (PPS). The PPS consisted of all the participants that complied with the study protocol, had no protocol deviation nor data missing at baseline, and had the major endpoints collected. The baseline characteristic of participants was analyzed in FAS. Efficacy analysis of primary and secondary endpoints (including the time to first negative viral testing, the time and rate of disappearance of clinical symptoms, and hospitalization duration) was applied to both FAS and PPS and showed robustness and consistency. The safety analysis including incidence of AEs, laboratory tests and vital signs after medication were applied in safety set (SS).

In this study, the primary endpoint was the time to first negative viral testing. Based on the one-sided sample size calculation with a given significance of 0.025 at the alpha-value level, 1-β power of 0.8, ratio of group sizes 1:1, design effect HR = 1.4, using the PASS software, the sample size of the Jin-Zhen Oral Liquid group was calculated as 110, and sample size of the positive control group was 110. Assuming the shedding rate of 10%, the sample size was designed as 120 in each group.

The Kaplan-Meier plot was used for examining the survival curve of time to first negative viral testing between two treatment groups; the log-rank test was used to compare the difference in the survival curve between the two groups, and COX regression was used to examine for covariates. The *t*-test or rank test was used for comparison of hospitalization duration. The *x* ^2^ test or Fisher’s exact was used to analyze the secondary endpoints including the rate of major clinical symptoms disappearance and incidence of conversion to the severe/critical type as appropriated method.

## Results

### Patients and clinical presentation

During the period from 2022.03.24 to 2022.05.25, a total of 240 patients were eligible for enrollment. 117 patients were assigned to Jin-Zhen Oral Liquid group and 123 patients were assigned to Jinhua Qinggan granules group (positive control group). Demographic and baseline clinical characteristics had no difference between the two groups (Table 1). The average age was 6.32 ± 3.52 years, and 134 (55.8%) children were male. Eight (3.3%) children had underlying diseases (double renal pelvis separation, tumor, asthma, Kawasaki’s disease, premature infants, pertussis like syndrome, and allergic asthma). The mean height of the children was 122.1 ± 25.0 cm and the mean body weight was 26.95 ± 13.57 kg. The symptoms of COVID-19 at baseline in both groups are shown in Supplementary Table S1. Fever was most common symptom in 87 children (74.4%) in the Jin-Zhen Oral Liquid group and 92 children (74.8%) in the positive control Jinhua Qinggan granules group, followed by dry cough in 57 (48.7%) and 47 (38.2%) in the Jin-Zhen Oral Liquid and positive control groups, respectively. All children completed the trial. One child in the Jin-Zhen Oral Liquid group taking a prohibited drug and two children in the Jinhua Qinggan granules group taking a prohibited drug, and they were not included in the PPS.

**TABLE 1:** Demographics and baseline characteristics of the subjects in the trial (FAS).

Variable		Jin-Zhen oral liquid group (n = 117)	Jinhua Qinggan granules group (n = 123)
*Demographics*			
Age (years)	mean (SD)	5.88(3.48)	6.73(3.53)
Gender (n,%)	Male	65(55.6)	69(56.1)
	Female	52(44.4)	54(43.9)
Height (cm)	mean (SD)	118.1(25.9)	125.9(23.5)
Weight (kg)	mean (SD)	24.99(12.84)	28.82(14.02)
Underlying disease (n,%)	Yes	111(94.9)	121(98.4)
	No	6(5.1)	2(1.6)
	Tumor	1(0.9)	0(0.0)
	others	5(4.3)	2(1.6)
*Laboratory examination*			
Hemoglobin (ng/dL)	median (min, max)	130.00 (92, 176)	134.00(98, 173)
Neutrophil count	median (min, max)	1.79 (0.3,6.9)	2.10 (0.3, 13.4)
Platelets/μL	median (min, max)	226.00 (118,453)	221.50 (100, 454)
Red blood cell count	median (min, max)	4.74(3.05, 6)	4.77(3.88, 5.69)
White blood cell count	median (min, max)	5.45 (2, 11.32)	4.94 (3.05, 552)
AST U/L	median (min, max)	27.00 (2, 57)	25.50 (6,278)
ALT U/L	median (min, max)	12.00 (6, 50)	10.00 (5, 152)
Cr	median (min, max)	30.50 (9, 53.4)	34.00 (16, 75)
Total serum bilirubin	median (min, max)	5.00 (1, 68)	5.15 (1.5, 38)
*Vital signs*			
SPO_2_	median (min, max)	99.00 (93, 100)	99.00(95, 100)
Respiratory rate	median (min, max)	23.00 (16, 40)	22.00(16, 32)
Body temperature	median (min, max)	36.60 (36, 39.5)	36.80 (36, 39.8)
Pulse	median (min, max)	100.00 (78, 142)	98.00 (74, 146)
Disease grade (n,%)	asymptomatic	5(4.3)	5(4.1)
	common	0(0.0)	5(4.1)
	mild	112(95.7)	113(91.9)

### Efficacy

#### Primary efficacy endpoint

**FIGURE 2:**
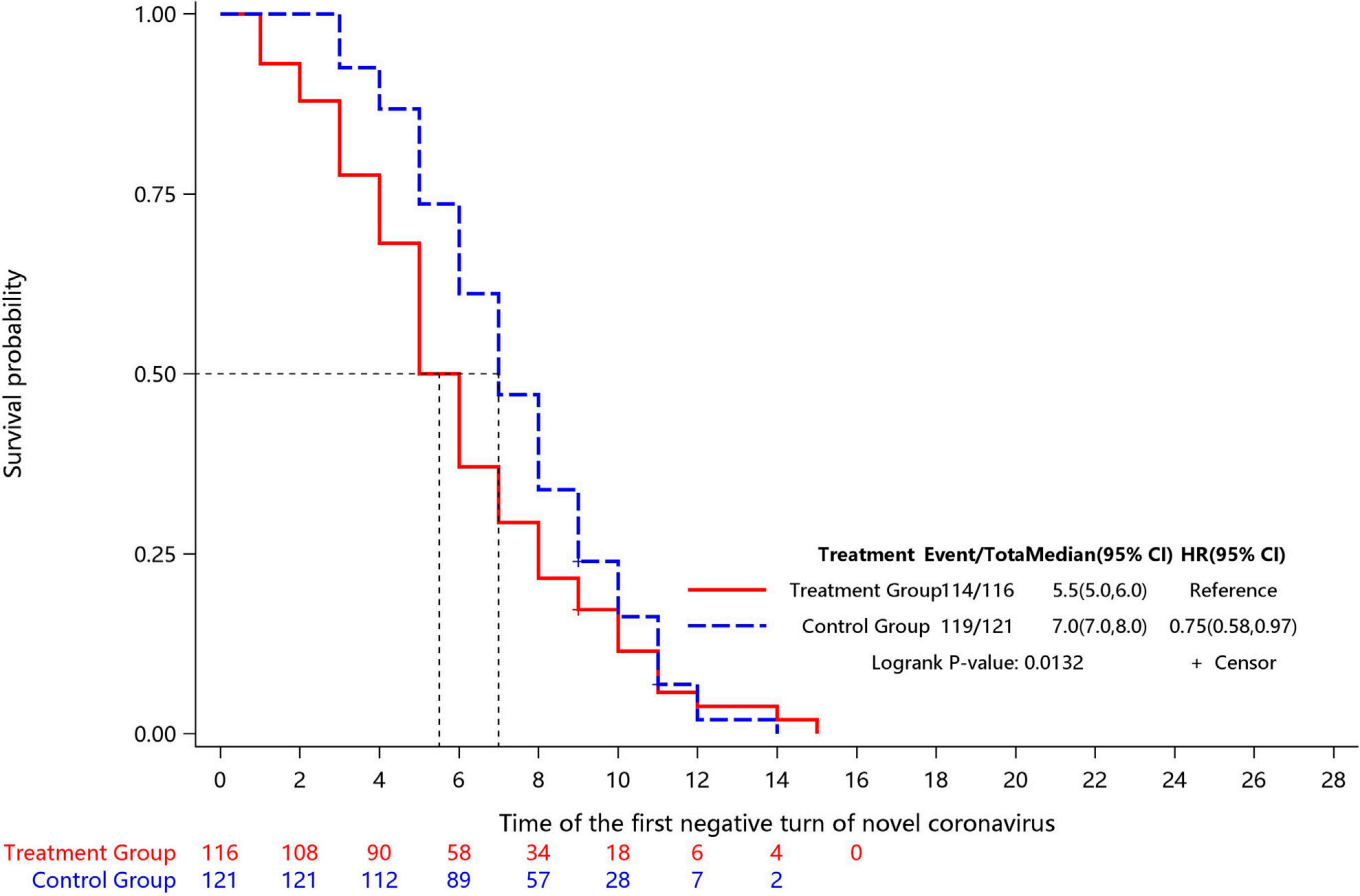
Kaplan-Meier survival curve of the time to first negative viral testing (FAS).

Two children in the Jin-Zhen Oral Liquid group and two children in the positive control Jinhua Qinggan granules group were missing the data of first time COVID-19 regression after treatment. The time to first negative viral testing was significantly shorter (0.76, 95% CI 0.59-0.98) in the Jin-Zhen Oral Liquid group (median 6.0 days, 95% CI 5.0-6.0) compared with the control group (median 7.0 days, 95% CI 7.0-8.0) (Table 2; Figure 2, Supplementary Table S2; Supplementary Figure S1).

**TABLE 2:** The time to first negative viral testing in two groups (FAS).

	Jin-Zhen oral liquid group (n = 117)	Jinhua Qinggan granules group (n = 123)
mean ± SD	6.1 ± 0.30	7.5 ± 0.24
median(95%CI)	6.0(5.0,6.0)	7.0(7.0,8.0)
Q1(95%CI)	4.0(3.0,5.0)	5.0(5.0,6.0)
Q3(95%CI)	8.0(7.0,10.0)	9.0(9.0,10.0)
log-rank *p*-value	0.0176	
HR(95%CI)	0.76(0.59,0.98)	
HR *p*-value	0.0377	

The time to first negative viral testing = The time of the first negative viral testing in the two consecutive negative viral testing (>24 h apart) - The time of first medication.

#### Secondary efficacy endpoints

The time of major clinical symptoms (including fever, dry cough, sputum, fatigue, vomiting, diarrhea, pharyngoxerosis, nasal congestion, and nasal discharge) disappearance was similar in two groups, with no significant difference (Table 3). However, Jin-Zhen Oral Liquid had a shorter time to improve the pharyngalgia symptoms of pediatric COVID-19 patients (median 4.0 days in Jin-Zhen Oral Liquid group vs. 6.5 days in the positive control group, *p* < 0.05). The rate of major clinical symptoms disappearance showed no significant difference between Jin-Zhen Oral Liquid compared to the positive control Jinhua Qinggan Granules group (Table 4). Furthermore, the hospitalization duration was significantly reduced in the Jin-Zhen Oral Liquid treatment group. The median hospitalization duration days for pediatric COVID-19 patients in the Jin-Zhen Oral Liquid group was 10.0 days, which was significantly shorter than that of the positive control group (median 12.0 days) (*p* = 0.0007) (Table 5). None of the pediatric COVID-19 patients in two groups had exacerbated to critical illness.

**TABLE 3:** The time to disappearance of major clinical symptoms in the two groups after treatment (FAS).

		Jin-Zhen oral liquid group (n = 117)	Jinhua Qinggan granules group (n = 123)	*p*-value
fever	n	87	92	
	mean ± SD	1.9 ± 1.3	1.9 ± 1.1	0.9163
	Median (min, max)	1.0(1.0, 7.0)	1.0(1.0, 5.0)	
dry cough	n	57	47	
	mean ± SD	6.4 ± 4.7	7.3 ± 5.8	0.3522
	Median (min, max)	5.5(1.0, 25.0)	7.0(1.0, 28.0)	
sputum	n	24	26	
	mean ± SD	5.9 ± 4.7	6.0 ± 3.3	0.9151
	Median (min, max)	6.0(1.0, 25.0)	6.0(1.0–14.0)	
fatigue	n	1	2	
	mean ± SD	2.0	1.5 ± 0.7	0.6667
	Median (min, max)	2.0(2.0, 2.0)	1.5(1.0, 2.0)	
vomiting	n	2	3	
	mean ± SD	1.5 ± 0.7	1.0 ± 0.0	0.2722
	Median (min, max)	1.5(1.0, 2.0)	1.0(1.0, 1.0)	
diarrhea	n	8	4	
	mean ± SD	2.6 ± 2.0	1.5 ± 0.6	0.305
	Median (min, max)	1.5(1.0, 5.0)	1.5(1.0, 2.0)	
Pharyngoxerosis	n	6	3	
	mean ± SD	2.8 ± 1.7	3.7 ± 2.5	0.5708
	Median (min, max)	3.0(1.0, 5.0)	4.0(1.0, 6.0)	
Pharyngalgia	n	25	28	
	mean ± SD	4.1 ± 2.4	5.7 ± 2.2	0.0147
	Median (min, max)	4.0(1.0, 10.0)	6.5(1.0, 10.0)	
nasal congestion	n	4	4	
	mean ± SD	3.0 ± 2.8	3.5 ± 4.4	0.8537
	Median (min, max)	1.0(1.0, 7.0)	1.5(1.0, 10.0)	
nasal discharge	n	3	7	
	mean ± SD	2.0 ± 1.0	2.7 ± 3.3	0.7268
	Median (min, max)	2.0(1.0, 3.0)	2.0(1.0, 10.0)	

**TABLE 4:** The rate of major symptoms disappearance at day7, 14 and 28 (FAS).

Major symptoms	Time	Jin-zhen oral liquid group (n = 117)	Jinhua Qinggan granules group (n = 123)	*p*-value
Fever				
Disappearance rate (%, 95%CI)	Day 7	100.0 (95.8, 100.0)	100.0 (96.1, 100.0)	-
Disappearance rate (%, 95%CI)	Day 14	100.0 (95.8, 100.0)	100.0 (96.1, 100.0)	-
Disappearance rate (%, 95%CI)	Day 28	100.0 (95.8, 100.0)	100.0 (96.1, 100.0)	-
Dry cough				
Disappearance rate (%, 95%CI)	Day 7	80.7 (68.1,90.0)	59.6(44.3,73.6)	0.0288
Disappearance rate (%, 95%CI)	Day 14	94.7(85.4,98.9)	93.6(82.5,98.7)	1.0
Disappearance rate (%, 95%CI)	Day 28	100.0 (93.7,100.0)	100.0 (92.5,100.0)	-
Sputum				
Disappearance rate (%, 95%CI)	Day 7	95.8 (78.9,99.9)	73.1 (52.2,88.4.0)	0.054
Disappearance rate (%, 95%CI)	Day 14	95.8 (78.9,99.9)	100.0 (86.8,100.0)	0.48
Disappearance rate (%, 95%CI)	Day 28	100.0 (85.8,100.0)	100.0 (86.8,100.0)	
Fatigue				
Disappearance rate (%, 95%CI)	Day 7	100.0 (2.5,100.0)	100.0 (15.8,100.0)	-
Disappearance rate (%, 95%CI)	Day 14	100.0 (2.5,100.0)	100.0 (15.8,100.0)	-
Disappearance rate (%, 95%CI)	Day 28	100.0 (2.5,100.0)	100.0 (15.8,100.0)	-
Vomiting				
Disappearance rate (%, 95%CI)	Day 7	100.0 (15.8,100.0)	100.0 (29.2,100.0)	-
Disappearance rate (%, 95%CI)	Day 14	100.0 (15.8,100.0)	100.0 (29.2,100.0)	-
Disappearance rate (%, 95%CI)	Day 28	100.0 (15.8,100.0)	100.0 (29.2,100.0)	-
Diarrhea				
Disappearance rate (%, 95%CI)	Day 7	100.0 (63.1,100.0)	100.0 (39.8,100.0)	-
Disappearance rate (%, 95%CI)	Day 14	100.0 (63.1,100.0)	100.0 (39.8,100.0)	-
Disappearance rate (%, 95%CI)	Day 28	100.0 (63.1,100.0)	100.0 (39.8,100.0)	-
pharyngoxerosis				
Disappearance rate (%, 95%CI)	Day 7	100.0 (54.1,100.0)	100.0 (29.2,100.0)	-
Disappearance rate (%, 95%CI)	Day 14	100.0 (54.1,100.0)	100.0 (29.2,100.0)	-
Disappearance rate (%, 95%CI)	Day 28	100.0 (54.1,100.0)	100.0 (29.2,100.0)	-
pharyngalgia				
Disappearance rate (%, 95%CI)	Day 7	84.0 (63.9,95.5)	50.0 (30.6,69.4)	0.0109
Disappearance rate (%, 95%CI)	Day 14	100.0 (86.3,100.0)	100.0 (87.7,100.0)	-
Disappearance rate (%, 95%CI)	Day 28	100.0 (86.3,100.0)	100.0 (87.7,100.0)	-
Nasal congestion				
Disappearance rate (%, 95%CI)	Day 7	100.0 (39.8,100.0)	75.0 (19.4,99.4)	1
Disappearance rate (%, 95%CI)	Day 14	100.0 (39.8,100.0)	100.0 (39.8,100.0)	-
Disappearance rate (%, 95%CI)	Day 28	100.0 (39.8,100.0)	100.0 (39.8,100.0)	-
Nasal discharge				
Disappearance rate (%, 95%CI)	Day 7	100.0 (29.2,100.0)	85.7 (42.1,99.6)	1
Disappearance rate (%, 95%CI)	Day 14	100.0 (29.2,100.0)	100.0 (59.0,100.0)	-
Disappearance rate (%, 95%CI)	Day 28	100.0 (29.2,100.0)	100.0 (59.0,100.0)	-

Disappearance rate of major clinical symptoms=(Number of patients with major clinical symptoms disappeared/total number of patients with major clinical symptoms at baseline)×100%.

**TABLE 5:** Hospitalization duration in the two groups after treatment (FAS).

		Jin-Zhen oral liquid group (n = 117)	Jinhua Qinggan granules group (n = 123)	*p*-value
hospitalization duration (days)	N (miss)	117(0)	123(0)	0.0007
	mean ± SD	10.4 ± 3.2	11.7 ± 2.9	
	Median (Q1∼Q3)	10.0(8.0–12.0)	12.0(10.0–14.0)	
	min, max	3, 18	5, 19	

Hospitalization duration = The time of discharge from hospital- The time of first medication.

#### Safety assessment

No serious adverse events were observed during the study. Among 240 children, 1 patient (0.8%) in the positive control group had twice adverse events (vomiting and facial eczema), neither of which were serious adverse events. No adverse events were observed in the Jin-Zhen Oral Liquid group (0.0%) (Table 6). No patient discontinued the trial due to adverse events. During the treatment period, there was no significant difference between the Jin-Zhen Oral Liquid group and the positive control group in laboratory tests after day 14 or at discharge, and the difference in vital signs was comparable (Supplementary Tables S3, S4).

**TABLE 6:** Adverse events (Safety set).

	Jin-Zhen oral liquid group (n = 117)		Jinhua Qinggan granules group (n = 123)	
	N (%)	Frequency	N (%)	Frequency
Any adverse events	0(0.0)	0	1(0.8)	2
Treatment Emergent Adverse Events (TEAE)	0(0.0)	0	1(0.8)	2
Serious adverse events during treatment	0(0.0)	0	0(0.0)	0
Serious drug-related adverse events during treatment	0(0.0)	0	0(0.0)	0
Adverse events during treatment leading to the death	0(0.0)	0	0(0.0)	0
Adverse events during treatment leading to discontinuation of trial regimen	0(0.0)	0	0(0.0)	0

## Discussion

In this study, we clarify that Jin-Zhen Oral Liquid is effective and safe for the treatment of mild to moderate COVID-19 in children. Jin-Zhen Oral Liquid has shortened the time to first negative viral testing compared to the positive control Jinhua Qinggan Granules. In addition, Jin-Zhen Oral Liquid shorten the hospitalization duration.

Traditional Chinese medicine has shown effect on the treatment of COVID-19 and rehabilitation (Huang et al., 2021). Systemic review of clinical trials show that traditional Chinese medicine is effective in the treatment and rehabilitation of COVID-19 (Wang et al., 2021; Kang et al., 2022). There are mainly three kinds of indicators in evaluating the effectiveness of treating COVID-19, including overall indicator (negative conversion of nucleic acids for the first time, hospitalization duration), symptom indicator (fever, cough, or feebleness) and laboratory examination indicator (white blood cells, lymphocytes and so on) (Yu et al., 2022). In the present study, the primary endpoint was time to first negative viral testing. The secondary endpoints were the time and rate of major symptoms disappearance, duration of hospitalization, and the proportion of symptoms changed from asymptomatic or mild to moderate or severe/critical illness. In addition, the safety end points of any adverse events were observed. In previous studies, Yindan Jiedu granules, Reduning injection and Shuanghuanglian oral liquids showed effective in reducing time to first negative viral testing (Ni et al., 2021; Xu et al., 2021; Feng et al., 2022), while Hua Shi Bai Du granule and Lianhuaqingwen capsules was failed to shorten the time of nucleic acid turning negative (Hu et al., 2021; Liu et al., 2021). Most of the clinical trials have shown the improvement of symptoms with traditional Chinese medicine (Yin et al., 2021). Patients treated with Reduning injection have shown shorter hospitalization duration (Ma et al., 2021). There were few clinical studies about the traditional Chinese medicine in the treatment of COVID-19 children. We found that Jin-Zhen Oral Liquid shortened the time to first negative viral testing, shortened the hospitalization duration and relived patient symptoms faster when compared with the positive control Jinhua Qinggan Granules.

In this study, Jinhua Qinggan granules was selected as the positive control administration. Jinhua Qinggan granules was the first proprietary Chinese medicine with significant efficacy against influenza A (H1N1) during the 2009 influenza A (H1N1) pandemic (Qi et al., 2017). It has been listed as a recommended medicine in the “Pneumonia treatment protocol for COVID-19 infection (Version 9)” jointly issued by the National Health and Welfare Commission and the State Administration of Traditional Chinese Medicine (National Health Commission and Medicine NAoTC, 2022). Jinhua Qinggan granules has shown effects in shortening the febrile period, reducing inflammation, and improving and treating symptoms associated with mild to moderate COVID-19 (Lu et al., 2010; Liu et al., 2020; An et al., 2021). A randomized, double-blind, placebo-controlled clinical study of 300 patients showed that Jinhua Qinggan granules significantly reduced the levels of leukocytes, ferritin, and CRP, shortened the median recovery time of symptoms associated with COVID-19 (including cough, sputum, sore throat, dyspnea, headache, nasal congestion, fatigue and myalgia), and show a low incidence of adverse events (3 mild to moderate adverse events) that were well tolerated by patients (Shah et al., 2022).

Jin-Zhen Oral Liquid has been marketed for more than 20 years and has showed well-established tolerability and safety. The common adverse effects include diarrhea, abdominal pain, stomach discomfort and rash. In this study, no adverse events have been observed in Jin-Zhen Oral Liquid treatment group. It suggests that Jin-Zhen Oral Liquid could be well-tolerated and safe treatment for children with COVID-19. The molecular mechanism of Jin-Zhen Oral Liquid for the treatment of COVID-19 in children is still unclear. COVID-19 belongs to the category of “epidemic diseases” in traditional Chinese medicine theory. In addition, COVID-19 is considered as a “cold and dampness epidemic”, which is consistent with the physiopathologic mechanism of blocking the lungs. In traditional Chinese medicine, “epidemic” refers to plague, as a general concept of severe infectious disorders. A screening of classical Chinese herbal formulas for the prevention and treatment of plague using a network pharmacology approach revealed that licorice, scutellaria, rhubarb and chai-hu contained compounds that could dovetail with the targets of COVID-19 (Ren et al., 2020). Jin-Zhen Oral Liquid contains three main compounds of them. Glycyrrhetinic acid, the main active component of licorice, is a potent binder of high mobility group protein B1 (HMGB1), which significantly reduces virus-induced inflammatory response and viral replication and alleviates respiratory distress syndrome associated with viral infection (Bailly and Vergoten, 2020), and also reduces IL-33 levels in serum and bronchoalveolar fluid (Fu et al., 2016). Glycyrrhetinic acid significantly inhibits lipopolysaccharide or interleukin-4 induced MUC5AC mRNA expression, inhibits mucus overproduction and airway epithelial cell inflammatory response, and acts as a cough suppressant (Nishimoto et al., 2010). Rhodopsin, the main active component of rhubarb, inhibits the interaction of the S-protein of SARS-CoV-2 with the ACE2 protein thereby inhibiting S-protein retrovirus infection. Since SARS-CoV-2 has a protein interaction conformation structurally like that of SARS-CoV-2, it is suggested that rhodopsin may also inhibit SARS-CoV-2 infection by blocking the interaction of the S-protein of SARS-CoV-2 with the human ACE2 protein through this pathway (Ho et al., 2007). Astragalus reduces the level of inflammatory transmitters, induces interferon to resist the virus, promotes cellular and humoral immunity, and exerts broad-spectrum antiviral effects (Huang et al., 2019). Astragalus polysaccharide, the main active component of Astragalus, can regulate the expression levels of interleukins (IL-12, IL-10), tumor cell necrosis factor (TNF-α mRNA) and proteins (Wang et al., 2011). Tao et al. used network pharmacology and molecular docking techniques to construct an herb-compound-target network between the chemical components of Jin-Zhen Oral Liquid and COVID-19 related target proteins, and found nine key compounds and 10 potentially acting target proteins, suggesting that Jin-Zhen Oral Liquid may regulate the expression of bromodomain containing protein 2 (Brd2), aminopeptidase N (CD13) and ACE2 by as well as interfering with PI3K/Akt, Jak-STAT, TNF and MAPK signaling pathways to contain the onset and development of COVID-19 cytokine storm (Zong et al., 2018; Tao et al., 2020).

This study has several limitations. First, the sample size was small. Further study with larger sample size might provide more clinical evidence. Second, we did not design the placebo group in this study. According to the Guidelines on Diagnosis and Treatment Protocol of novel coronavirus pneumonia (COVID-19) in China, traditional Chinese medicine was recommended for COVID-19 patients. Therefore, this study was designed as a positive comparator non-inferiority study, but not placebo-controlled trial. We conducted this trial in using Jinhua Qinggan granules as a positive comparator. Third, this trial was not designed in a double-blind and double-dummy trial. Because the production of liquid and granules, taste, favor, color were different from the Jin-Zhen Oral Liquid and the positive control (Jinhua Qinggan granules), this study was conducted as an open-label trial. However, the primary and secondary endpoints such as the time to first negative viral testing, the duration of hospitalization were objective indicators.

In this study, we investigated the non-inferiority effect of Jin-Zhen Oral Liquid compared to the established anti-COVID-19 agent Jinhua Qinggan granules as the positive comparator. Jin-Zhen Oral Liquid is non-inferior to Jinhua Qinggan granules in shortening the time to first negative viral testing, the time and rate of major clinical symptoms relief, and hospitalization duration. It is safe and effective in the treatment of mild to medium COVID-19 in children. It is expected to become a promising therapeutic strategy for pediatric COVID-19 patients.

## Data Availability

The original contributions presented in the study are included in the article/Supplementary Material, further inquiries can be directed to the corresponding authors.

## References

[B1] Alsohime F. Temsah M. H. Al-Nemri A. M. Somily A. M. Al-Subaie S. (2020). COVID-19 infection prevalence in pediatric population: Etiology, clinical presentation, and outcome. J. Infect. Public Health 13 (12), 1791–1796. 10.1016/j.jiph.2020.10.008 33127335 PMC7574780

[B2] An X. Xu X. Xiao M. Min X. Lyu Y. Tian J. (2021). Efficacy of Jinhua qinggan granules combined with western medicine in the treatment of confirmed and suspected COVID-19: A randomized controlled trial. Front. Med. 8, 728055. 10.3389/fmed.2021.728055 PMC8545827 34712679

[B3] Bailly C. Vergoten G. (2020). Glycyrrhizin: An alternative drug for the treatment of COVID-19 infection and the associated respiratory syndrome? Pharmacol. Ther. 214, 107618. 10.1016/j.pharmthera.2020.107618 32592716 PMC7311916

[B4] Children and COVID-19 (2022). State-level data report. Available from: https://www.aap.org/en/pages/2019-novel-coronavirus-covid-19-infections/children- and-covid-19-state-level-data-report/ .

[B5] FDA (2022). Fact sheet for healthcare providers: Emergency use authorization for paxlovid. Available from: https://www.fda.gov/media/155050/download .

[B6] Feng Y. Zhu B. Liu Y. Zhou G. Yang L. (2022). Yindan Jiedu granules exhibit anti-inflammatory effect in patients with novel Coronavirus disease (COVID-19) by suppressing the NF-κB signaling pathway. Phytomedicine 95, 153784. 10.1016/j.phymed.2021.153784 34785108 PMC8484181

[B7] Fu J. Lin S. H. Wang C. J. Sheng-Yuan L. Feng X. Y. Liu Q. (2016). HMGB1 regulates IL-33 expression in acute respiratory distress syndrome. Int. Immunopharmacol. 38, 267–274. 10.1016/j.intimp.2016.06.010 27318792

[B8] Ho T. Y. Wu S. L. Chen J. C. Li C. C. Hsiang C. Y. (2007). Emodin blocks the SARS coronavirus spike protein and angiotensin-converting enzyme 2 interaction. Antivir. Res. 74 (2), 92–101. 10.1016/j.antiviral.2006.04.014 16730806 PMC7114332

[B9] Hu K. Guan W. J. Bi Y. Zhang W. Li L. Zhang B. (2021). Efficacy and safety of lianhuaqingwen capsules, a repurposed Chinese herb, in patients with coronavirus disease 2019: A multicenter, prospective, randomized controlled trial. Phytomedicine 85, 153242. 10.1016/j.phymed.2020.153242 33867046 PMC7229744

[B10] Huang K. Zhang P. Zhang Z. Youn J. Y. Wang C. Zhang H. (2021). Traditional Chinese Medicine (TCM) in the treatment of COVID-19 and other viral infections: Efficacies and mechanisms. Pharmacol. Ther. 225, 107843. 10.1016/j.pharmthera.2021.107843 33811957 PMC8011334

[B11] Huang Y. Du Z. C. Hou X. T. Hao E. W. Qin H. Z. Deng J. G. (2019). Astragali Radix residues. Residues. Chem. Compos. Pharmacol. Action Appl. Res. Prog. inChinese J. Inf. TCM 26 (6), 140–144.

[B12] Kang X. Jin D. Jiang L. Zhang Y. Zhang Y. An X. (2022). Efficacy and mechanisms of traditional Chinese medicine for COVID-19: A systematic review. Chin. Med. 17 (1), 30. 10.1186/s13020-022-00587-7 35227280 PMC8883015

[B13] Liu J. Yang W. Liu Y. Lu C. Ruan L. Zhao C. (2021). Combination of hua shi Bai Du granule (Q-14) and standard care in the treatment of patients with coronavirus disease 2019 (COVID-19): A single-center, open-label, randomized controlled trial. Phytomedicine 91, 153671. 10.1016/j.phymed.2021.153671 34425471 PMC8285932

[B14] Liu J. Zhang G. L. Huang G. Q. Li L. Li C. P. Wang M. (2014). Therapeutic effect of jinzhen oral liquid for hand foot and mouth disease: A randomized, multi-center, double-blind, placebo-controlled trial. PLoS One 9 (4), e94466. 10.1371/journal.pone.0094466 24722423 PMC3983189

[B15] Liu Z. Li X. Gou C. Li L. Luo X. Zhang C. (2020). Effect of Jinhua Qinggan granules on novel coronavirus pneumonia in patients. J. Tradit. Chin. Med. 40 (3), 467–472. 10.19852/j.cnki.jtcm.2020.03.016 32506862

[B16] Lu Q. Bao Y. X. Wang W. Xie X. T. Yu J. F. Chen Y. (2010). A prospective multicenter randomized controlled clinical study on the efficacy and safety of Guaifenesin compound pseudoephedrine hydrochloride oral solution. Chin. J. Pract. Pediatr. 48 (3), 204–207. 20426957

[B17] Ma Q. Wang Z. Chen R. Lei B. Liu B. Jiang H. (2022). Effect of Jinzhen granule on two coronaviruses: The novel SARS-CoV-2 and the HCoV-229E and the evidences for their mechanisms of action. Phytomedicine 95, 153874. 10.1016/j.phymed.2021.153874 34923232 PMC8665848

[B18] Ma Q. Xie Y. Wang Z. Lei B. Chen R. Liu B. (2021). Efficacy and safety of ReDuNing injection as a treatment for COVID-19 and its inhibitory effect against SARS-CoV-2. J. Ethnopharmacol. 279, 114367. 10.1016/j.jep.2021.114367 34174375 PMC8223030

[B19] National Health Commission and Medicine NAoTC (2022). Diagnosis and treatment protocol for novel coronavirus pneumonia (Trial Version 9). Available from: http://www.nhc.gov.cn/yzygj/s7653p/202203/b74ade1ba4494583805a3d2e40093d88. shtml 133:1087-95.

[B20] Ni L. Wen Z. Hu X. Tang W. Wang H. Zhou L. (2021). Effects of shuanghuanglian oral liquids on patients with COVID-19: A randomized, open-label, parallel-controlled, multicenter clinical trial. Front. Med. 15 (5), 704–717. 10.1007/s11684-021-0853-6 33909260 PMC8079840

[B21] NIH (2022). Remdesivir. Available from: https://www.covid19treatmentguidelines.nih.gov/therapies/antiviral-therapy/remdesivir/ .

[B22] Nishimoto Y. Hisatsune A. Katsuki H. Miyata T. Yokomizo K. Isohama Y. (2010). Glycyrrhizin attenuates mucus production by inhibition of MUC5AC mRNA expression *in vivo* and *in vitro* . J. Pharmacol. Sci. 113 (1), 76–83. 10.1254/jphs.09344fp 20453436

[B23] Qi Y. Gao F. Hou L. Wan C. (2017). Anti-inflammatory and immunostimulatory activities of astragalosides. Am. J. Chin. Med. 45 (6), 1157–1167. 10.1142/S0192415X1750063X 28830214

[B24] Ren X. Shao X. X. Li X. X. Jia X. H. Song T. Zhou W. Y. (2020). Identifying potential treatments of COVID-19 from Traditional Chinese Medicine (TCM) by using a data-driven approach. J. Ethnopharmacol. 258, 112932. 10.1016/j.jep.2020.112932 32376368 PMC7196535

[B25] Shah M. R. Fatima S. Khan S. N. Ullah S. Himani G. Wan K. (2022). Jinhua qinggan granules for non-hospitalized COVID-19 patients: A double-blind, placebo-controlled, and randomized controlled trial. Front. Med. (Lausanne) 9, 928468. 10.3389/fmed.2022.928468 35979216 PMC9376460

[B26] Tao X. Q. Ke Z. P. Zhang X. Z. Deng Y. Cao Z. Y. Cao L. (2020). Investigate Mechanism of jinzhen oral liquid for prevention COVID-19 based on network pharmacology. Chin. Tradit. Herb. Drugs 51, 2326–2333.

[B27] Wang C. Cao B. Liu Q. Q. Zou Z. Q. Liang Z. A. Gu L. (2011). Oseltamivir compared with the Chinese traditional therapy maxingshigan-yinqiaosan in the treatment of H1N1 influenza: A randomized trial. Ann. Intern Med. 155 (4), 217–225. 10.7326/0003-4819-155-4-201108160-00005 21844547

[B28] Wang Z. Li L. Song M. Yan J. Shi J. Yao Y. (2021). Evaluating the traditional Chinese medicine (TCM) officially recommended in China for COVID-19 using ontology-based side-effect prediction framework (OSPF) and deep learning. J. Ethnopharmacol. 272, 113957. 10.1016/j.jep.2021.113957 33631276 PMC7899032

[B29] Xu X. Zhang J. Zheng W. Yang Z. Zhao X. Wang C. (2021). Efficacy and safety of reduning injection in the treatment of COVID-19: A randomized, multicenter clinical study. Ann. Palliat. Med. 10 (5), 5146–5155. 10.21037/apm-20-2121 33894725

[B30] Yin B. Bi Y. M. Sun L. Huang J. Z. Zhao J. Yao J. (2021). Efficacy of integrated traditional Chinese and western medicine for treating COVID-19: A systematic review and meta-analysis of RCTs. Front. Public Health 9, 622707. 10.3389/fpubh.2021.622707 34307269 PMC8298033

[B31] Yu R. Zhang S. Zhao D. Yuan Z. (2022). A systematic review of outcomes in COVID-19 patients treated with Western medicine in combination with traditional Chinese medicine versus Western medicine alone. Expert Rev. Mol. Med. 24, e5. 10.1017/erm.2021.35 34986905 PMC8795778

[B32] Zong S. B. Sun L. Lv Y. Z. Zhou J. Wang Z. Z. Xiao W. (2018). Effects of jinzhen oral liquid on NF-κb and MAPK signaling pathway in LPS-induced acute lung injury model mice. China J. Exp. Tradit. Med. formulae. 13 (9), 155–159.

