# Supplementary figures and images for: Jin-Zhen oral liquid for pediatric coronavirus disease (COVID-19): A randomly controlled, open-label, and non-inferiority trial at multiple clinical centers
Source: Front Pharmacol. 2023 Feb 27;14:1094089. doi: 10.3389/fphar.2023.1094089 (PMC10008848; doi:10.3389/fphar.2023.1094089)

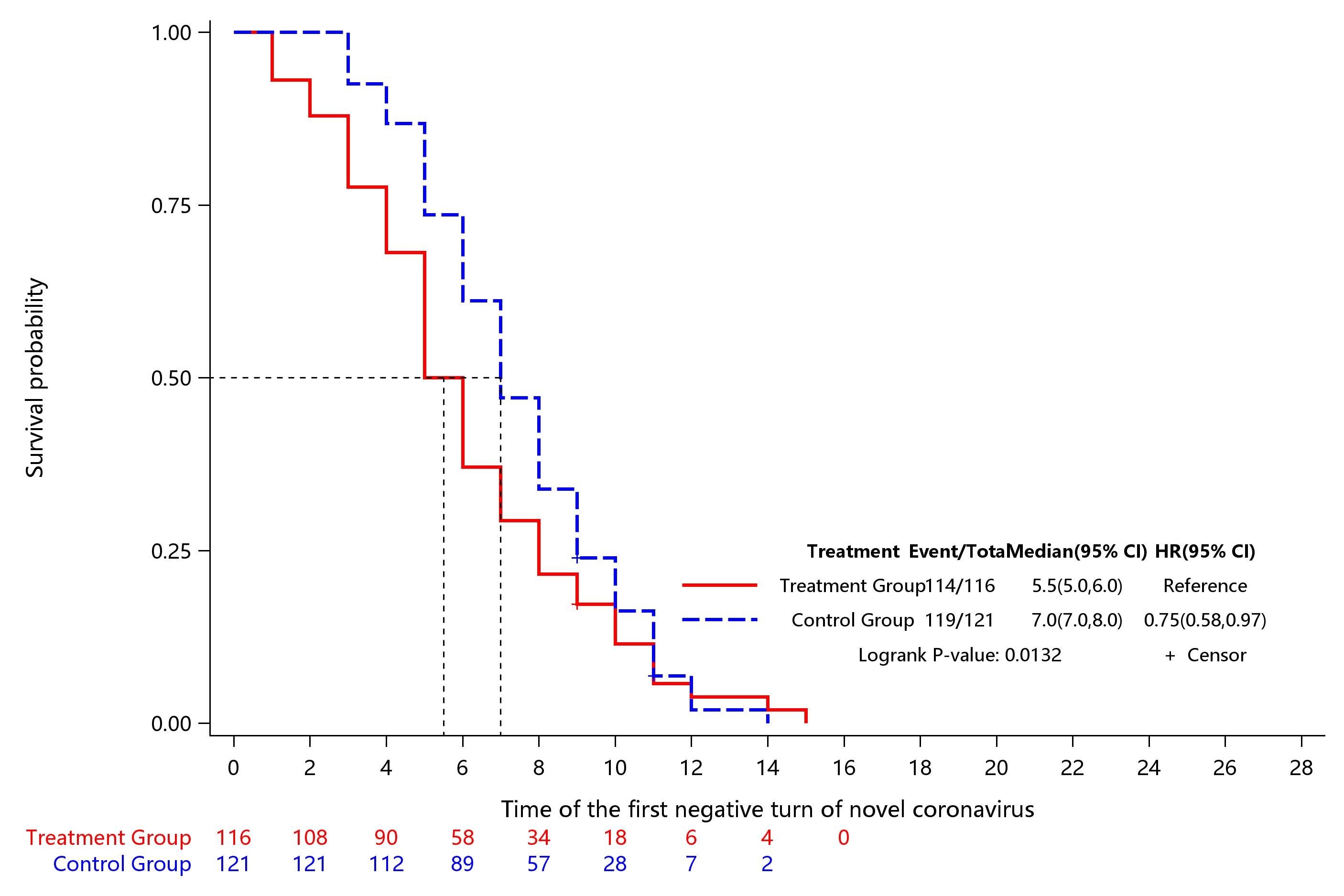

Supplement: Supplementary file 1 [file Image1.tiff]
