# Supplementary material for: Jin-Zhen oral liquid for pediatric coronavirus disease (COVID-19): A randomly controlled, open-label, and non-inferiority trial at multiple clinical centers
Source: Front Pharmacol. 2023 Feb 27;14:1094089. doi: 10.3389/fphar.2023.1094089 (PMC10008848; doi:10.3389/fphar.2023.1094089)
Supplement: Supplementary file 2 [file DataSheet1.pdf]

Supplement Tables

TABLE S1. Initial symptoms in the two groups

		Jin-Zhen Oral Liquid group (n=117)	Jinhua Qinggan granules group (n=123)	Total(n=240)
fever (n,%)	Yes	87(74.4)	92(74.8)	179(74.6)
	No	29(24.8)	31(25.2)	60(25.0)
	Absence	1(0.9)	0(0.0)	1(0.4)
dry cough (n,%)	Yes	57(48.7)	47(38.2)	104(43.3)
	No	59(50.4)	76(61.8)	135(56.3)
	Absence	1(0.9)	0(0.0)	1(0.4)
sputum (n,%)	Yes	24(20.5)	26(21.1)	50(20.8)
	No	92(78.6)	97(78.9)	189(78.8)
	Absence	1(0.9)	0(0.0)	1(0.4)
fatigue (n,%)	Yes	1(0.9)	2(1.6)	3(1.3)
	No	115(98.3)	121(98.4)	236(98.3)
	Absence	1(0.9)	0(0.0)	1(0.4)
vomiting (n,%)	Yes	2(1.7)	3(2.4)	5(2.1)
	No	114(97.4)	120(97.6)	234(97.5)
	Absence	1(0.9)	0(0.0)	1(0.4)
diarrhea (n,%)	Yes	8(6.8)	4(3.3)	12(5.0)
	No	108(92.3)	119(96.7)	227(94.6)
	Absence	1(0.9)	0(0.0)	1(0.4)
poor mental response (n,%)	No	116(99.1)	123(100.0)	239(99.6)
	Absence	1(0.9)	0(0.0)	1(0.4)
Shortness of breath (n,%)	No	116(99.1)	123(100.0)	239(99.6)
	Absence	1(0.9)	0(0.0)	1(0.4)
Pharyngoxerosis (n,%)	Yes	6(5.1)	3(2.4)	9(3.8)
	No	110(94.0)	120(97.6)	230(95.8)
	Absence	1(0.9)	0(0.0)	1(0.4)
Pharyngalgia (n,%)	Yes	25(21.4)	28(22.8)	53(22.1)
	No	91(77.8)	95(77.2)	186(77.5)
	Absence	1(0.9)	0(0.0)	1(0.4)

		Jin-Zhen Oral Liquid group (n=117)	Jinhua Qinggan granules group (n=123)	Total(n=240)
nasal congestion (n,%)	Yes	4(3.4)	4(3.3)	8(3.3)
	No	112(95.7)	119(96.7)	231(96.3)
	Absence	1(0.9)	0(0.0)	1(0.4)
Nasal discharge (n,%)	Yes	3(2.6)	7(5.7)	10(4.2)
	No	113(96.6)	116(94.3)	229(95.4)
	Absence	1(0.9)	0(0.0)	1(0.4)
chest discomfort (n,%)	No	116(99.1)	123(100.0)	239(99.6)
	Absence	1(0.9)	0(0.0)	1(0.4)
emotional stress (n,%)	No	116(99.1)	123(100.0)	239(99.6)
	Absence	1(0.9)	0(0.0)	1(0.4)
anorexia (n,%)	Yes	1(0.9)	0(0.0)	1(0.4)
	No	115(98.3)	123(100.0)	238(99.2)
	Absence	1(0.9)	0(0.0)	1(0.4)

TABLE S2. The time to first negative viral testing in two groups (PPS)

	Jin-Zhen Oral Liquid group (n=117)	Jinhua Qinggan granules group (n=123)
mean±SD	6.1±0.30	7.5±0.24
median(95%CI)	5.5(5.0,6.0)	7.0(7.0,8.0)
Q1(95%CI)	4.0(3.0,5.0)	5.0(5.0,6.0)
Q3(95%CI)	8.0(7.0,9.0)	9.0(9.0,10.0)
log-rank P value	0.0132	
HR（95%CI)	0.75(0.58,0.97)	
HR P value	0.03	

The time to first negative viral testing = The time of the first negative viral testing in the two consecutive negative viral testing (>24 h apart) - The time of first medication

TABLE S3. Laboratory tests on the 14th day after medication in the two groups (SS)

Laboratory examination		Jin-Zhen Oral Liquid group	Jinhua Qinggan granules group
Hemoglobin (ng/dL)	n(nmiss)	93(24)	112(11)
	median (min, max)	134.00(106, 169)	134.00(104, 169)
Neutrophil count	n(nmiss)	76(41)	83(40)
	median (min, max)	2.35(0.6, 6.61)	2.20(0.8, 18)
Platelets /μL	n(nmiss)	93(24)	112(11)
	median (min, max)	326.00(31, 584)	310.00(3.5, 491)
Red blood cell count	n(nmiss)	93(24)	112(11)
	median (min, max)	4.80(3.25, 6.03)	4.81(3.72, 5.82)
White blood cell count	n(nmiss)	93(24)	112(11)
	median (min, max)	6.39(3.04, 12.05)	6.01(3.32, 10.56)
AST U/L	n(nmiss)	56(61)	68(55)
	median (min, max)	24.00(2, 60)	22.50(12, 37)
ALT U/L	n(nmiss)	56(61)	68(55)
	median (min, max)	13.00(6, 43)	11.00(4, 49)
Cr	n(nmiss)	56(61)	68(55)
	median (min, max)	28.00(12, 51)	32.00(11, 67)
Total serum bilirubin	n(nmiss)	56(61)	68(55)
	median (min, max)	6.15(2.4, 13.5)	5.80(2.7, 18.8)

ALT, alanine aminotransferase. AST, aspartate aminotransferase. Cr, creatinine.

TABLE S4. Vital signs on the 14th day after medication in the two groups (SS)

vital sign		Jin-Zhen Oral Liquid group	Jinhua Qinggan granules group
SPO_2	n(nmiss)	20(97)	31(92)
	median (min, max)	99.00(98.00, 100.00)	99.00(97.00, 100.00)
Respiratory rate	n(nmiss)	21(96)	31(92)
	median (min, max)	23.00(17.00, 30.00)	22.00(16.00, 28.00)
Body temperature	n(nmiss)	21(96)	31(92)
	median (min, max)	36.50(36.30, 36.80)	36.50(36.00, 36.90)
Pulse	n(nmiss)	21(96)	31(92)
	median (min, max)	98.00(78.00, 994.00)	90.00(70.00, 116.00)
